# Physical activity and active transportation behaviour among rural, peri-urban and urban children in Kenya, Mozambique and Nigeria: The PAAT Study

**DOI:** 10.1371/journal.pone.0262768

**Published:** 2022-01-21

**Authors:** Lucy-Joy Wachira, Sylvester O. Hayker, Richard Larouche, Adewale L. Oyeyemi, Antonio Prista, George E. Owino, Mark S. Tremblay, Vincent O. Onywera

**Affiliations:** 1 Department of Physical Education, Exercise and Sport Science, Kenyatta University, Nairobi Kenya; 2 Department of Hospitality and Leisure Studies, The Technical University of Kenya, Nairobi, Kenya; 3 Faculty of Health Sciences, University of Lethbridge, Lethbridge, Canada; 4 Department of Physiotherapy, University of Maiduguri, Maiduguri, Nigeria; 5 Physical Activity and Health Research Group, Research Centre on Physical Activity and Sports, Universidade Pedagogica, Maputo, Mozambique; 6 Department of Sociology, Gender and Development Studies, Kenyatta University, Nairobi, Kenya; 7 Healthy Active Living and Obesity Research Group, Children’s Hospital of Eastern Ontario Research Institute, Ottawa, Canada; 8 Department of Physical Education, Exercise and Sport Science, Kenyatta University, Nairobi, Kenya; La Inmaculada Teacher Training Centre (University of Granada), SPAIN

## Abstract

**Background:**

Physical activity (PA) is associated with numerous health benefits among children and youth. However, few studies have examined how active transportation (AT) and device-based measures of PA vary within and between countries in sub-Saharan Africa.

**Purpose:**

This cross-sectional study sought to investigate the prevalence and correlates of AT and device-measured PA among children living in urban, peri-urban and rural areas in three African countries representing Eastern, Western and Southern regions of Africa.

**Methods:**

3,205 participants (53.3% girls; 46.7% boys) aged 10–12 years were recruited in Kenya, Nigeria and Mozambique. Data were collected using a child questionnaire, a parent/guardian questionnaire and PiezoRx® pedometers. ANCOVA and binary logistic regression analyses were used to examine the correlates of AT and PA while controlling for gender, age, parent education and vehicle ownership.

**Results:**

Participants accumulated an average of 45.6±23.5 min/day of moderate-to-vigorous physical activity (MVPA) and 11,215±4,273 steps/day. Kenyan and Mozambican children were significantly more active than their Nigerian counterparts (*p*<0.001). Only 23% met the MVPA guidelines of 60 min/day. 65.1% of participants engaged in AT to school (and 67.8% for the trip back home) with no gender differences. Living in a rural area, lower parent education, lower vehicle ownership and higher motorcycle ownership were associated with higher odds of AT. Other correlates of AT were country-specific. Girls accumulated less daily MVPA than boys in all countries. MVPA was positively associated with living in less urbanized areas in Nigeria and Mozambique. In Kenya, lower parental education and AT were associated with higher MVPA. Nigerian children’s daily MVPA decreased with age and the number of parent-perceived barriers to AT.

**Conclusions:**

Majority of children engaged in AT, but still failed to meet MVPA recommendations. Most correlates of AT and PA were country-specific, suggesting that strategies to encourage both behaviours should be informed by local evidence.

## Introduction

Physical activity (PA) in childhood is associated with numerous immediate and long-term health benefits [1]. There is evidence that PA behaviour tracks through adolescence and into adulthood [2]. All intensity levels, including light intensity PA, are beneficial for health although higher levels of intensity such as moderate-to-vigorous intensity PA (MVPA) offer greater health benefits [3]. It is recommended that children and adolescents should accumulate an average of at least 60 minutes/day of MVPA across the week [4]. However, global trends indicate that majority of children and adolescents are insufficiently active [5–7]. Data from African countries indicate that the prevalence of insufficient PA among school going adolescents in Africa was 86.2%, ranging from 75.9% in Benin to 90.3% in Sudan [5, 8]. Among many health problems, insufficient MVPA is associated with the development of cardiovascular disease risk factors in childhood and adolescence [9].

There is insufficient and inconclusive data on the prevalence of PA among children in Africa, and especially studies with device-measured PA data [10]. A systematic review of studies that used both self-reported and device-based measures found a large discrepancy in children’s PA [11]. Self-reported measures are subject to social desirability and recall biases, which may lead to an overestimation of PA levels [11]. Some studies from low-income countries (LICs) have also found that PA was lower among urban residents compared to rural residents [12–14]. These observations are consistent with the PA transition model, which posits that urbanization and economic growth are associated with the adoption of a sedentary lifestyle that tends to happen in urban before rural areas [15]. Because of the rapid urbanization and high rates of rural to urban migration in many LICs [16], the potential shift towards an even more sedentary lifestyle is of concern.

Consistent evidence indicates that children and youth who engage in active transportation (AT; the use of non-motorized travel modes such as walking, running and cycling) to and from school are more active than those who use motorized travel modes [17]. In developing countries such as those in Africa, walking and cycling tend to remain the main modes of transportation for supporting many activities of daily living among the general population [18]. In the African context, a large proportion of overall PA is thought to be accumulated through the use of non-motorized travel modes or AT [19]. Furthermore, AT is an inexpensive form of PA that can help reduce vehicle emissions that contribute to climate change and cardio-respiratory diseases [20].

A systematic review concluded that few studies have examined travel patterns and their correlates among African children [17]. The best available evidence indicates that AT is less common in urban areas and among children attending higher socioeconomic status schools [17, 18]. Nationally-representative data from 15 African countries indicate that between 33.6% and 66.6% of youth reported walking or cycling to/from school at least once a week [21]. In another study, Peltzer [22] reported that between 19.8% and 31.1% of 13- to 15-year-olds from Kenya, Uganda, Zambia and Zimbabwe reported engagement in AT to and/from school at least 5 days/week. A review of literature from developing countries found consistent evidence that the prevalence of AT is higher in rural versus urban areas, among children from poorer families, and those who live closer to school [18]. However, evidence for other potential correlates was deemed insufficient. Also, no studies have specifically examined how AT and device-based measures of PA and their correlates vary across the levels of urbanization within and between countries in sub-Saharan Africa (SSA).

A better understanding of the correlates of AT among African children could help inform the development of interventions aimed at preventing a potential shift towards motorized travel modes. Decreasing rates of PA and AT may contribute to an increased risk of non-communicable diseases especially in countries of Africa where health systems are constrained by limited resources. There is still paucity of data and adequate, conclusive and accurate information on childhood AT and PA from SSA countries limiting the creation of collective public health strategies and interventions. Additionally, there are very few multi-country studies conducted in urban, suburban, and rural areas of Sub-Saharan Africa (SSA) using objective measures of PA and with a large sample size. Some of the previous studies on the PA transition involved a few dozen participants in urban and rural areas, and suburban areas are often excluded. Studies also heavily rely on self-report methods. The few existing studies on AT among children in SSA have also focused on trips to only one destination (school) and engaged a shorter monitoring period (less than 7 days of device wear). Our study sought to address these research gaps and presents important new information, especially on unique multicounty and regional differences, which is necessary for ongoing PA research in SSA. The aim of this study was to investigate how individual, household, and environmental correlates of AT and pedometer-measured PA among children living in urban, peri-urban, and rural areas vary within and between three African countries. To the best of our knowledge, this has not been studied previously. Countries were selected to represent the Eastern, Western and Southern regions of Africa to increase the generalizability of the findings to other countries in Sub-Saharan Africa.

## Methods

This study was part of a larger research project entitled, “The Physical Activity and Active Transport (PAAT) project” that employed a cross-sectional design. The PAAT project was a multi-country study assessing physical activity and active transportation among school children aged 10–12 years in Eastern (Kenya), Western (Nigeria) and Southern (Mozambique) regions of Africa. The study also focused on a comparison between urban, peri-urban and rural settings. The study protocol was reviewed and approved by institutional review boards across the three countries (Kenyatta University Ethics Review Committee in Kenya, National Health Bioethics Committee of Mozambique, the Nigeria Heart Foundation Ethics Committee in Nigeria) and the Children’s Hospital of Eastern Ontario Research Institute in Canada where collaborators were affiliated. Relevant research permits were obtained prior to data collection. Written Informed consent was obtained from parents/guardians and written assent was obtained from participating children.

The study was conducted in Nairobi and Kisumu counties in Kenya, in Maputo city and Bobole (Marracuene District) in Mozambique and in Lagos state in Nigeria. We aimed to recruit a gender-balanced sample of 1,000 participants from each country with an equal distribution of participants across regions (urban, peri-urban and rural). Schools were purposely selected to access participants aged 10–12 years in each region. A class-based sampling approach was used to identify the most appropriate class/grade with the targeted age group and the best gender and age distribution. The age of the participants was also confirmed from their date of birth as reported by their parent/guardian. Exclusion criteria included the presence of injuries, illnesses or conditions that could restrict AT and PA.

Data collection instruments included a child questionnaire, a parent/guardian questionnaire and a pedometer [19]. The questionnaires were closed-ended with prewritten response categories. The questionnaires were translated from English to local languages in the three countries [Kenya (*Kiswahili*), Nigeria (*Yoruba*) and Mozambique (*Portuguese*)] and back-translated to ensure that the meaning of the questions was preserved. Translation was done by professional translators and the back-translation was done by bilingual researchers who also have an understanding of the cultural context. Both the child and parent/guardian questionnaires included items on the mode used by the children to get to school and perceived barriers to active transportation [19]. School travel mode was assessed with the items “*How does your child usually go to school in a typical week (from Monday to Friday)*?” and “*How does your child usually go back home from school in a typical week (from Monday to Friday)*?”. Response options included walking, biking, running, car/van, bus/train, motorcycle, and other. Twenty items (S1 Table) assessing environmental, psychosocial, planning, and safety barriers to AT were adapted from a US study by Forman et al. [23] to the African context. During pilot-testing, we observed that participants struggled with the original 4-point scale from strongly disagree to strongly agree (with “somewhat” agree and disagree options in the middle). Therefore, we changed response options to “yes” and “no” to facilitate comprehension. The parent/guardian questionnaire also collected data on sociodemographic characteristics of the household. Parent education was assessed with the item “*what is the highest level of education achieved by any of the mother/father/guardian in this home*?” and eight options ranging from less than primary school to graduate degree were offered. Parents were also asked to report the number of functioning vehicles and motorcycles in their household on a scale from 0 to 4 or more.

The PiezoRx® (StepsCounts, Deep River, Canada) pedometer was used to objectively assess the number of step counts/day and minutes of MVPA. The estimation of MVPA was based on validated cut-points of 110 steps/minute for moderate- and 130 steps/minute for vigorous-intensity PA [24]. Children were instructed to wear the pedometer on their waist or hip for seven consecutive days during waking hours. Once the device was collected from the participant, data were retrieved and recorded into an Excel data sheet for further data treatment (described below).

Prior to data collection, a familiarization session was held with the research team to review administrative procedures, assess feasibility and logistics, practice use of research tools, data collection, data management, quality control and data entry. The reliability and validity of the questionnaires were also verified [19].

Data collection was done through schools where participants were given pedometers (and a verbal explanation on how to wear it) and the questionnaire. They were also given the parents’ questionnaire to deliver to their parent/guardian (and return once complete).

### Data treatment

Pedometer data were treated in accordance with the rules and procedures recommended by Rowe et al. [25]. Specifically, we considered pedometer data to be “valid” if participants had at least 3 days (including one weekend day) where between 1,000 and 29,999 steps were recorded. We replaced excluded days with the mean of “valid” weekdays or weekend days, as appropriate. We then calculated the average number of steps per day [25]. If step counts for a given day were outside the 1,000–29,999 range, the MVPA data for that day were considered “invalid,” and we employed the same approach to replace the MVPA data. We calculated average MVPA/day by dividing the minutes of MVPA by the number of “valid” days. We recoded parent education as “less than high school”, “some high school” or “tertiary” (i.e., college or university) based on the observed frequency distribution. Similarly, we recategorized the number of vehicles and motorcycles as “0”, “1”, and “2 or more”. For inferential statistics, we dichotomized the child’s mode of transportation to school reported by parents as active vs. motorized. We calculated indices of child- and parent-perceived barriers to AT based on the 20 barriers (S1 Table) that were coded as yes (1) or no (0), with higher scores indicating a greater number of perceived barriers. For descriptive purposes, we assessed compliance with the WHO [4] MVPA guidelines in two different ways: 1) via the above-mentioned step frequency thresholds to determine MVPA of ≥60 min/day; and 2) as the proportion of children who accumulated at least 12,000 steps/day on average. The latter threshold was found to provide the best approximation of adherence to the MVPA guidelines based on step counts [26].

### Data analysis

Descriptive statistics were used to examine school travel mode, step counts, MVPA, socio-demographic characteristics of the household and perceived barriers to AT. All analyses were stratified by country. Chi-square tests and one-way analyses of variance (ANOVA) with Tukey post-hoc tests were used to examine differences between countries. Binary logistic regression models were used to examine the association between predictors (gender, age, type of urbanization, parent education and the number of cars/trucks and motorcycles in the household, and perceived barriers to AT) and school travel mode. A similar approach was used to examine the correlates of steps/day using analysis of covariance (ANCOVA) models, except we centred continuous variables at the grand mean [27]. In both binary logistic regression and ANCOVA models, gender, age, type of urbanization, and parent education were deemed mandatory variables to control for sampling variables and household socio-economic status. They were also retained in all multivariable models, except in Mozambique where parent education was not available. We used a backward selection method to remove other variables that were not associated with outcome variables at *p*<0.05. All statistical analyses were conducted using the Statistical Package for Social Sciences (IBM SPSS^®^, Armonk, New York) programme version 26 and missing data were deleted listwise. We assessed the proportion of explained variance with R^2^ for ANCOVA models and Nagelkerke’s pseudo-R^2^ for binary logistic regression models.

## Results

### Characteristics of participants and differences between countries

Descriptive statistics of the sample are provided in Table 1. There was a total of 3,205 participants (53.3% girls aged 11.1±0.9 years): 1,122 from Kenya, 1,097 from Mozambique, and 986 from Nigeria. Data on parent education and ownership of vehicles and motorcycles were not available for Mozambique. Parents in Nigeria reported higher levels of education and higher car/truck ownership while Kenyan parents reported higher levels of motorcycle ownership (all *p*<0.001). Overall, 65.1% of participants engaged in AT to school and 67.8% engaged in AT from school. The prevalence of AT was much higher in Kenya and Mozambique compared to Nigeria (*p*<0.001).

**Table 1:** Descriptive characteristics of the sample stratified by country.

Study Variable		Kenya		Mozambique		Nigeria	
		Frequency	Percent	Frequency	Percent	Frequency	Percent
Gender	Boy	520	46.3	482	43.9	494	50.1
	Girl	602	53.7	615	56.1	492	49.9
Region	Urban	398	35.5	344	31.5	320	32.5
	Peri-urban	372	33.2	390	35.7	369	37.4
	Rural	352	31.4	359	32.8	297	30.1
Parent education level	Primary & below	380	33.9	-	-	179	18.4
	High School	443	39.5	-	-	380	39.1
	Tertiary	298	26.6	-	-	414	42.5
Number of cars/trucks	0	518	46.2	-	-	423	43.3
	1	463	41.3	-	-	358	36.6
	2 or more	141	12.6	-	-	196	20.1
Number of motorcycles	0	530	47.2	-	-	721	74.1
	1	491	43.8	-	-	187	19.2
	2 or more	101	9.0	-	-	65	6.7
School travel mode	Walking	835	74.4	706	70.7	315	32.9
	Cycling	3	0.3	0	0.0	54	5.6
	Running	88	7.8	1	0.1	0	0.0
	Car/van	137	12.2	292	29.2	113	11.8
	Bus/train	55	4.9	0	0.0	263	27.5
	Motorcycle	4	0.4	0	0.0	211	22.1
Home travel mode	Walking	875	78.0	681	71.8	387	40.5
	Cycling	4	0.4	0	0.0	45	4.7
	Running	62	5.5	1	0.1	0	0.0
	Car/van	120	10.7	267	28.1	73	7.6
	Bus/train	54	4.8	0	0.0	237	24.8
	Motorcycle	7	0.6	0	0.0	214	22.4

Table 2 shows descriptive statistics for steps/day, average time per day in MVPA, and perceived barriers to AT. A total of 2,840 participants (88.6% of the sample) provided valid pedometer data. These participants accumulated an average of 45.6±23.5 min/day of MVPA and 11,215±4,273 steps/day. Overall, 23.0% of participants achieved an average of 60 min/day of MVPA based on the pedometer MVPA threshold whereas 40.7% met the guideline based on the 12,000 steps/day threshold. One-way ANOVA analyses indicated that Kenyan children accumulated significantly more steps/day than Mozambican children who were more active than Nigerian children. The proportion of children who met the MVPA guidelines was similar in Kenya and Mozambique (29.2% and 29.6% respectively), but lower in Nigeria (9.5%; χ^2^_[2 df]_ = 139.1; *p*<0.001). When using the 12,000 steps/day threshold, 53.8% of Kenyan, 45.5% of Mozambican and 20.4% of Nigerian children met the guidelines with significant differences between countries (χ^2^_[2 df]_ = 244.0; *p*<0.001). There were no statistically significant differences in the average time spent in MVPA between Kenyan and Mozambican children who were both more active (on average) than their Nigerian counterparts (*p*<0.001). Mozambican parents perceived the highest number of barriers to AT followed by Nigerian and Kenyan parents. Nigerian children perceived more barriers to AT than their Kenyan counterparts.

**Table 2:** Differences between countries in measures of PA and perceived barriers to AT.

Variable	Country	N	Mean (SD)	F	p	Post-hoc test
Steps/day	All	2840	11215 (4,273)	206.78	< .001	-
	Kenya	1120	12508 (3,669)	-	-	>M >N
	Mozambique	804	11895 (4,430)	-	-	>N <K
	Nigeria	916	9036 (3,968)	-	-	<K <M
MVPA (min/day)	All	2840	45.6 (23.5)	135.59	< .001	-
	Kenya	1120	50.7 (21.3)	-	-	>N
	Mozambique	804	50.0 (26.8)	-	-	>N
	Nigeria	916	35.5 (19.6)	-	-	<K <M
Parent perceived barriers to AT	All	3065	6.8 (3.6)	73.55	< .001	-
	Kenya	1098	6.0 (3.2)	-	-	<M <N
	Mozambique	1023	7.8 (3.7)	-	-	>K >N
	Nigeria	944	6.6 (3.8)	-	-	>K <M
Child perceived barriers to AT	All	2103	6.6 (3.4)	81.69	< .001	-
	Kenya	1122	5.9 (3.2)	-	-	<N
	Mozambique	-	-	-	-	-
	Nigeria	981	7.2 (3.4)	-	-	>K

Note: AT: active transportation; MVPA: moderate- to vigorous-intensity physical activity. The post-hoc column shows significant differences between countries (K = Kenya; M = Mozambique; N = Nigeria) based on Tukey post-hoc tests where letters represent the first letter of the comparison countries.

### Correlates of active transportation

Tables 3 and 4 show the correlates of AT in bivariate and multi-variable analyses. In all countries, gender was not associated with the likelihood of AT and children living in less urbanized areas were more likely to engage in AT. In Mozambique, 100% of children living in rural areas and 99.1% of those living in peri-urban areas engaged in AT. Among Kenyan and Nigerian children, lower parental education and owning less than 2 cars were generally associated with greater odds of AT. However, car/truck ownership was not significant for the trip back home in Nigeria. Nigerian children whose family owned fewer motorcycles were less likely to engage in AT. The odds of AT increased by 33–37% with each year of age in Nigeria, but age was not associated with AT in Kenya or Mozambique. In Nigeria, each additional child- or parent-perceived barrier to AT was independently associated with a 7% decrease in the odds of AT to school, with similar results for the trip back home (Table 4). In contrast, each additional barrier to AT perceived by Mozambican parents was associated with an 8–9% increase in the odds of AT (Table 3). This was the only significant correlate of AT in Mozambican children. Correlates of school travel mode were generally consistent for the trip to school and back home.

**Table 3:** Results of bivariate models examining the correlates of AT for the trip to school and home from school.

	Trip to school						Trip home from school					
	Kenya		Mozambique		Nigeria		Kenya		Mozambique		Nigeria	
Variable	OR (95% CI)	*p*	OR (95% CI)	*p*	OR (95% CI)	*p*	OR (95% CI)	*p*	OR (95% CI)	*p*	OR (95% CI)	*p*
Gender (girl)	0.96 (0.70–1.31)	.783	0.91 (0.69–1.20)	.522	1.13 (0.87–1.46)	.374	0.98 (0.71–1.35)	.896	0.95 (0.72–1.27)	.736	1.16 (0.91–1.51)	.231
Gender (boy–ref)	-	-	-	-	-	-	-	-	-	-	-	-
Age (year)	1.17 (0.97–1.41)	.111	0.91 (0.79–1.06)	.227	**1.20 (1.02–1.42)**	**.029**	1.21 (0.99–1.46)	.058	0.91 (0.78–1.05)	.201	**1.19 (1.01–1.39)**	**.038**
Region (urban–ref)	-	-	-	-	-	-	-	-	-	-	-	-
Region (peri-urban)	**1.53 (1.07–2.18)**	**.021**	-	-	1.26 (0.91–1.76)	.169	**1.32 (0.92–1.90)**	**.138**	-	-	1.04 (0.76–1.42)	.813
Region (rural)	**2.31 (1.55–3.45)**	**< .001**	-	-	**2.98 (2.12–4.18)**	**< .001**	**2.31 (1.52–3.52)**	**< .001**	-	-	**2.75 (1.97–3.84)**	**< .001**
Parent education (less than high school)	**8.67 (5.43–13.85)**	**< .001**	-	-	**2.89 (2.00–4.16)**	**< .001**	**8.31 (5.16–13.38)**	**< .001**	-	-	**2.25 (1.57–3.23)**	**< .001**
Parent education (some high school)	**4.06 (2.82–5.82)**	**< .001**	-	-	**1.62 (1.20–2.18)**	**.002**	**4.40 (3.02–6.42)**	**< .001**	-	-	1.27 (0.95–1.68)	.106
Parent education (tertiary–ref)	-	-	-	-	-	-	-	-	-	-	-	-
No. of cars/trucks (0)	**8.06 (4.99–13.04)**	**< .001**	-	-	**2.07 (1.44–2.95)**	**< .001**	**6.34 (3.93–10.22)**	**< .001**	-	-	1.31 (0.93–1.84)	.127
No. of cars/trucks (1)	**2.00 (1.34–3.00)**	**< .001**	-	-	0.96 (0.65–1.40)	.811	**2.03 (1.34–3.07)**	**.001**	-	-	0.80 (0.56–1.14)	.222
No. of cars/trucks (2 or more–ref)	-	-	-	-	-	-	-	-	-	-	-	-
No. of motorcycles (0)	0.97 (0.53–1.76)	.912	-	-	**0.47 (0.28–0.80)**	**.005**	0.93 (0.51–1.72)	.823	-	-	**0.51 (0.30–0.87)**	**.013**
No. of motorcycles (1)	0.68 (0.38–1.23)	.204	-	-	**0.49 (0.27–0.87)**	**.015**	0.73 (0.40–1.34)	.305	-	-	**0.54 (0.30–0.96)**	**.035**
No. of motorcycles (2 or more–ref))	-	-	-	-	-	-	-	-	-	-	-	-
Parent-perceived barriers to AT (each unit increase)	1.04 (0.99–1.09)	.162	**1.08 (1.04–1.13)**	**< .001**	**0.91 (0.88–0.95)**	**< .001**	1.04 (0.98–1.09)	.136	**1.09 (1.05–1.14)**	**< .001**	**0.89 (0.86–0.93)**	**< .001**
Child-perceived barriers to AT (each unit increase)	0.98 (0.94–1.03)	.440	-	-	**0.91 (0.87–0.94)**	**< .001**	1.00 (0.95–1.05)	.889	-	-	**0.90 (0.87–0.94)**	**< .001**

Note: ref: reference group.

**Table 4:** Correlates of travel mode to school stratified by country in multivariable analyses.

	Trip to school				Trip home from school			
	Kenya		Nigeria		Kenya		Nigeria	
Variable	OR (95% CI)	*p*	OR (95% CI)	*P*	OR (95% CI)	*p*	OR (95% CI)	*p*
Gender (girl)	0.83 (0.59–1.19)	.316	1.14 (0.86–1.52)	.375	0.87 (0.61–1.25)	.463	1.21 (0.92–1.60)	.375
Gender (boy–ref)	-	-	-	-	-	-	-	-
Age (year)	1.11 (0.89–1.38)	.361	**1.37 (1.13–1.66)**	**.001**	1.16 (0.93–1.45)	.194	**1.33 (1.11–1.60)**	**.002**
Region (urban–ref)	-	-	-	-	-	-	-	-
Region (peri-urban)	0.68 (0.38–1.21)	.186	**1.67 (1.15–2.42)**	**.007**	0.65 (0.37–1.15)	.140	1.37 (0.96–1.95)	.079
Region (rural)	**1.84 (1.16–2.92)**	**.010**	**3.39 (2.30–4.99)**	**< .001**	**1.87 (1.16–3.03)**	**.010**	**3.00 (2.06–4.35)**	**< .001**
Parent education (less than high school)	**5.98 (3.65–9.81)**	**< .001**	**2.00 (1.32–3.03)**	**.001**	**5.98 (3.63–9.87)**	**< .001**	**1.77 (1.18–2.65)**	**.006**
Parent education (some high school)	**2.96 (2.00–4.39)**	**< .001**	1.30 (0.93–1.82)	.124	**3.40 (2.26–5.09)**	**< .001**	1.13 (0.82–1.56)	.450
Parent education (tertiary–ref)	-	-	-	-	-	-	-	-
No. of cars/trucks (0)	**18.05 (9.23–35.30)**	**< .001**	**2.12 (1.40–3.21)**	**< .001**	**12.34 (6.35–23.99)**	**< .001**	1.31 (0.88–1.95)	.177
No. of cars/trucks (1)	**2.36 (1.48–3.76)**	**< .001**	0.97 (0.63–1.47)	.872	**2.29 (1.43–3.69)**	**.001**	0.84 (0.56–1.24)	.373
No. of cars/trucks (2 or more–ref)	-	-	-	-	-	-	-	-
No. of motorcycles (0)	**0.23 (0.11–0.49)**	**< .001**	**0.46 (0.25–0.82)**	**.009**	**0.28 (0.13–0.61)**	**.001**	**0.54 (0.31–0.97)**	**.040**
No. of motorcycles (1)	0.55 (0.28–1.07)	.079	**0.35 (0.18–0.67)**	**.002**	0.60 (0.31–1.19)	.147	**0.49 (0.26–0.94)**	**.030**
No. of motorcycles (2 or more–ref)	-	-	-	-	-	-	-	-
Parent-perceived barriers to AT (each unit increase)	-	-	**0.93 (0.89–0.98)**	**.003**	-	-	**0.91 (0.87–0.95)**	**< .001**
Child-perceived barriers to AT (each unit increase)	-	-	**0.93 (0.88–0.98)**	**.007**	-	-	**0.94 (0.89–0.99)**	**.018**

Note: ref: reference group. Trip to school: Nagelkerke R^2^ for Kenya = 0.291; Nigeria = 0.195.

Trip home from school: Nagelkerke R^2^ for Kenya = 0.264; Nigeria = 0.167.

For Mozambique, the univariate model between region and travel mode could not produce meaningful results because 100% of rural and 99.1% of peri-urban Mozambican children engaged in AT. Gender and age (Table 3) were not significantly associated with AT, and data on parent education and vehicle ownership were not collected. Mozambique was therefore excluded from the multi-variable models presented in Table 4.

### Correlates of physical activity

The correlates of steps/day and average daily MVPA in bivariate and multi-variable analyses are shown in Tables 5 and 6. Parent education and car/truck ownership were not associated with steps/day in any country. In Kenya and Mozambique, girls were less active than boys and age was not associated with steps/day. Kenyan children who engaged in AT to school had more steps/day than motorized travellers. Mozambican children who lived in rural and peri-urban areas had significantly more steps/day than their urban counterparts while the opposite was observed in Kenya. Among Nigerian children, neither gender nor type of urbanization were associated with steps/day in multi-variable models (Table 6). In Nigeria, step counts decreased with age and were lower among children who engaged in AT to school. Child- and parent-perceived barriers to AT were not associated with step counts in any country.

**Table 5:** Correlates of average daily step counts and average daily MVPA stratified by country in bivariate models.

	Average daily step counts						Average daily MVPA					
	Kenya		Mozambique		Nigeria		Kenya		Mozambique		Nigeria	
Variable	β (95% CI)	*p*	β (95% CI)	*P*	β (95% CI)	*P*	β (95% CI)	*p*	β (95% CI)	*p*	β (95% CI)	*p*
Gender (girl)	**-978 (-1405; -550)**	**< .001**	**-2340 (-2940; -1740)**	**< .001**	-293 (-807; 222)	.265	**-4.7 (-7.2; -2.2)**	**< .001**	**-12.2 (-15.9; -8.6)**	**< .001**	**-3.2 (-5.7; -0.7)**	**.013**
Gender (boy–ref)	-	-	-	-	-	-	-	-	-	-	-	-
Age (year)	239 (-27; 505)	.078	-12 (-345; 321)	.942	**-855 (-1173; -537)**	**< .001**	0.4 (-1.2; 1.9)	.626	-1.3 (-3.3; 0.7)	.205	**-2.8 (-4.3; -1.2)**	**.001**
Region (urban–ref)	-	-	-	-	-	-	-	-	-	-	-	-
Region (peri-urban)	**-573 (-1092; -54)**	**.031**	**2170 (1462; -2878)**	**< .001**	**787 (170; 1404)**	**.012**	-1.1 (-4.2; 1.9)	.460	**14.1 (9.8; 18.4)**	**< .001**	**4.4 (1.4; 7.4)**	**.005**
Region (rural)	-487 (-1013; 39)	.070	**2986 (2234; 3738)**	**< .001**	352 (-297; 1001)	.288	-1.9 (-4.9; 1.2)	.230	**16.9 (12.4; 21.5)**	**< .001**	**4.3 (1.1; 7.5)**	**.008**
Parent education (less than high school)	417 (-140; 974)	.142	-	-	-364 (-1088; 360)	.324	**5.6 (2.4; 8.8)**	**.001**	-	-	-2.9 (-6.5; 0.7)	.111
Parent education (some high school)	205 (-334; 744)	.456	-	-	535 (-38; 1107)	.067	2.5 (-0.6; 5.6)	.115	-	-	-0.3 (-3.1; 2.5)	.844
Parent education (tertiary–ref)	-	-	-	-	-	-	-	-	-	-	-	-
Number of cars/trucks (0)	417 (-267; 1100)	.232	-	-	-78 (-773; 617)	.826	2.6 (-1.4; 6.6)	.200	-	-	-2.5 (-5.9; 0.9)	.151
Number of cars/trucks (1)	616 (-76; 1308)	.081	-	-	334 (-383; 1050)	.361	3.6 (-0.5; 7.6)	.084	-	-	-0.6 (-4.1; 3.0)	.749
Number of cars/trucks (2 or more–ref)	-	-	-	-	-	-	-	-	-	-	-	-
Number of motorcycles (0)	413 (-367; 1194)	.299	-	-	**1058 (94; 2106)**	**.048**	1.8 (-2.8; 6.3)	.443	-	-	-0.4 (-5.6; 4.7)	.866
Number of motorcycles (1)	**830 (45; 1615)**	**.038**	-	-	1149 (-15; 2313)	.053	2.9 (-1.7; 7.5)	.214	-	-	-0.8 (-6.6; 4.9)	.778
Number of motorcycles (2 or more–ref)	-	-	-	-	-	-	-	-	-	-	-	-
School travel mode (active)	**1033 (470; 1597)**	**< .001**	**2147 (1461; 2833)**	**< .001**	**-1562 (-2086; -1037)**	**< .001**	**6.2 (2.9; 9.4)**	**< .001**	**13.9 (9.8; 18.0)**	**< .001**	0.3 (-2.3; 3.0)	.816
School travel mode (motorized–ref)	-	-	-	-	-	-	-	-	-	-	-	-
Parent-perceived barriers to AT (each unit increase)	-18 (-87; 50)	.599	6 (-106; 117)	.919	10 (-63; 82)	.788	-0.3 (-0.7; 0.1)	.202	0.3 (-0.2; 0.8)	.235	**-0.6 (-0.9; -0.2)**	**.001**
Child-perceived barriers to AT (each unit increase)	-18 (-86; 49)	.597	-	-	-63 (-141; 16)	.117	-0.2 (-0.6; 0.2)	.329	-	-	**-1.0 (-1.4; -0.7)**	**< .001**

Note: ref: reference group. Semicolons are used to separate the lower and upper limits of the confidence intervals for physical activity models.

**Table 6:** Correlates of average daily step counts and average daily MVPA stratified by country in multi-variable models.

	Average daily step counts						Average daily MVPA					
	Kenya		Mozambique		Nigeria		Kenya		Mozambique		Nigeria	
Variable	β (95% CI)	*p*	β (95% CI)	*p*	β (95% CI)	*P*	β (95% CI)	*p*	β (95% CI)	*p*	β (95% CI)	*p*
Intercept	12452 (11820; 13084)	< .001	11210 (7678; 14742)	< .001	9132 (8523; 9742)	< .001	53.2 (49.8; 56.6)	< .001	46.8 (43.0; 50.6)	< .001	35.9 (33.0; 38.8)	< .001
Gender (girl)	**-980 (-1408; -553)**	**< .001**	**-2302 (-2892; -1714)**	**< .001**	-256 (-764; 251)	.322	**-4.9 (-7.4; -2.4)**	**< .001**	**-11.5 (-15.1; -7.9)**	**< .001**	**-3.0 (-5.5; -0.5)**	**.021**
Gender (boy–ref)	-	-	-	-	-	-	-	-	-	-	-	-
Age (year)	154 (-112; 420)	.257	37 (-276; 349)	.816	**-742 (-1073; -411)**	**.001**	-0.0 (-1.6; 1.5)	.979	37 (-276; 349)	.816	**-2.2 (-3.9; -0.6)**	**.007**
Region (urban–ref)	-	-	-	-	-	-	-	-	-	-	-	-
Region (peri-urban)	**-609 (-1124; -94)**	**.020**	**1910 (1217; 2603)**	**< .001**	536 (-95; 1166)	.096	-1.5 (-4.5; 1.5)	.315	**12.4 (8.1; 16.6)**	**< .001**	**3.6 (0.5; 6.7)**	**.023**
Region (rural)	**-657 (-1182; -113)**	**.014**	**3132 (2395; 3868)**	**< .001**	536 (-141; 1213)	.120	-2.7 (-5.8; 0.4)	.081	**17.6 (13.0; 22.1)**	**< .001**	**3.4 (0.2; 6.7)**	**.040**
Parent education (less than high school)	220 (-360; 799)	.457	-	-	17 (-713; 748)	.963	**4.6 (1.2; 8.0)**	**.007**	-	-	-2.6 (-6.2; 0.9)	.148
Parent education (some high school)	151 (-404; 705)	.593	-	-	**758 (193; 1323)**	**.009**	2.1 (-1.1; 5.4)	.196	-	-	-0.4 (-3.2; 2.4)	.771
Parent education (tertiary–ref)	-	-	-	-	-	-	-	-	-	-	-	-
School travel mode (active)	**1025 (431; 1618)**	**.001**	-	-	**-1557 (-2094; -1019)**	**< .001**	**5.0 (1.5; 8.4)**	**.005**	-	-	-	-
School travel mode (motorized–ref)	-	-	-	-	-	-						
Child-perceived barriers to AT (each unit increase)	-	-	-	-	-	-	-	-	-	-	**-1.0 (-1.4; -0.7)**	**< .001**

Note: ref: reference group. For daily step counts: Model adjusted R^2^ = 0.032 for Kenya; 0.145 for Mozambique; 0.067 for Nigeria. For MVPA: Model adjusted R^2^ = 0.027 for Kenya; 0.118 for Mozambique; 0.051 for Nigeria.

Girls accumulated less daily MVPA than boys in all countries. Age was not associated with MVPA, except for Nigerian children whose daily MVPA decreased with age. In Nigeria and Mozambique, MVPA was positively associated with living in less urbanized areas. Lower parental education was associated with children’s MVPA in Kenya, but not Nigeria. AT to school was associated with higher MVPA in Kenya and Mozambique. Child- and parent-perceived barriers to AT were negatively associated with MVPA in Nigeria; however, only child perceived barriers to AT showed significance in the multi-variable model. Table 6 indicates a notable difference between correlates of MVPA and step counts, especially in Nigeria.

## Discussion

This study aimed to investigate PA and school travel behaviors of children in Kenya, Nigeria and Mozambique. We found that the majority of participants engaged in AT to and from school, but still failed to meet MVPA recommendations. The likelihood of engaging in AT was consistently higher among children living in rural areas and in households with lower vehicle ownership and parental education. Nigerian children were much less likely to engage in AT than their Kenyan and Mozambican counterparts and they accumulated less PA. The study also observed that many correlates of AT and PA were country-specific.

### Active transportation

Overall, we observed that about two thirds of participants engaged in AT to and from school, with substantial differences between countries. Similarly, previous African studies have reported significant differences ranging from 19.8% in Namibia [22] to 80% in Zimbabwe [28]. In our study, the prevalence of AT to school ranged from 38.5% in Nigeria to 82.5% in Kenya, and varied substantially between urban, peri-urban and rural areas within countries. Collectively, this body of evidence suggests that there are differences between (and likely within) countries in variables that can influence travel mode choice, which may include access to motor vehicles, distance, and level of urbanization, among others. The lower prevalence of AT in our Nigerian sample may reflect the high rate of motorization in the state of Lagos [29] where data were collected.

Children living in rural areas were more likely to engage in AT in all countries. For example, all Mozambican children living in rural areas and almost all those living in peri-urban areas (99.1%) engaged in AT to and from school. This finding is consistent with previous research in developing countries and the PA transition model [12, 13, 15, 18, 30, 31]. Even after adjusting for region, lower vehicle ownership was associated with higher odds of AT to school, except for the trip back home in Nigeria. Higher parental education was also consistently associated with lower odds of AT. Parental education may act as a proxy for income and affordability of vehicles. In the African context, driving a vehicle is commonly perceived as a sign of prestige and prosperity. Hence, car owners may drive irrespective of the distance to their destination and road congestion [32]. Motorized travel may also be a strategy to avoid dangers on the route to/from school. We found that children were less likely to engage in AT if their family owned fewer motorcycles. In Kenya and Mozambique, less than 1% of children travelled by motorcycle, so families who own motorcycles may not use them for chauffeuring children to/from school.

Interestingly, the relationship between perceived barriers to AT and the odds of engaging in AT varied markedly between countries. In Kenya, there were no associations, even in bivariate models. In Nigeria, both child- and parent-perceived barriers were independently associated with lower odds of AT in multi-variable models. However, Mozambican children were more likely to engage in AT if their parents perceived *more* barriers. The need vs. choice framework proposed by Salvo et al. [33] could help interpret these findings. They suggested that in low-income countries, individuals may need to engage in AT irrespective of safety concerns due to a lack of alternatives. In contrast, active travellers in high-income countries may be more likely to engage in AT by choice, especially in the absence of major perceived barriers. While all countries in our study were low- or middle-income countries, Nigerian families in Lagos may have had higher income and greater access to vehicles than their Mozambican counterparts. Alternatively, the severity of perceived barriers may matter more than the number of barriers, and our index measured the latter. Qualitative studies have highlighted concerns associated with violence, rape, robbery, traffic, wild animals, and the risk of drowning while fording rivers on the route to/from school in the rainy season as salient perceived barriers to AT among African parents and children [34–38].

We found no relationships between gender and the likelihood of AT in any country. Previous studies in low- and middle-income countries have rarely found substantial gender differences [21, 22, 39]. Age was not associated with AT in Kenya and Mozambique, but the odds of AT increased by 37% with each year of age in Nigeria. Previous research in high-income countries suggests that older children are more likely to use AT than their younger counterparts due to lower parental safety concerns and reduced (perceived) need for parental supervision [40, 41]. However, the lack of access to other travel modes may override safety concerns. We found that the prevalence of AT was marginally higher for the trip back home. Such a pattern is common in high-income countries where parents can conveniently drive their children to school on the way to work [42]. When parents escort their children to school, they tend to select the mode that is quickest and easiest for them [43]. African parents who have access to vehicles may also be more likely to drive their child on the way to work or in an effort to reduce lateness to school. There is also a notable increase in the use of commercial motorcycle transportation in most African settings [44] which can help in avoiding lateness. This may partly explain the lower use of AT to school compared to the trip back home.

### Physical activity

We found that only 23% of participants achieved the recommended average of 60 minutes of daily MVPA [4] based on validated step frequency thresholds. The proportion of children who met the MVPA guidelines was similar in Kenya and Mozambique, but lower in Nigeria. Kenyan children accumulated significantly more steps/day than Mozambican children who, in turn, were more active than Nigerian children. Previous findings from African countries indicate that only 8% to 35% of African school children engaged in MVPA for ≥60 min on at least 5 days per week [21]. We believe that the lower PA among Nigerian children may be explained by unique characteristics in the study location, including a higher number of barriers related to traffic and personal safety and a lack of safe places for outdoor play, but this speculation requires further investigation. When using the 12,000 steps/day threshold as an approximation of compliance to MVPA guidelines, 40.7% of children were considered sufficiently active. The higher prevalence suggests that African children may accumulate a large proportion of their PA through locomotion at intensities below MVPA (e.g., AT and household chores), especially in rural areas [19, 45].

Girls were less active than boys, except for step counts in Nigeria. This is consistent with previous research [5, 14, 46–49] and underscores the need for additional efforts to promote PA in girls. We also found that age was not associated with MVPA and steps/day except among the Nigerian children, whose daily MVPA and step/day decreased significantly with age. Similar age-related declines in PA have been reported elsewhere [46, 50–52].

### Correlates of physical activity

In general, the correlates of PA varied substantially between countries and PA indicators (steps/day versus MVPA), suggesting that the correlates of PA may be context- and measurement-specific. For example, we found that the relationship between PA and urbanization did not consistently support the PA transition model [15]. Specifically, children living in less urbanized areas in Nigeria and Mozambique accumulated more MVPA and Mozambican children living in rural and peri-urban areas had significantly more steps/day than their urban counterparts. Unexpectedly, the opposite was observed in Kenya, where children living in urban areas were more active than their rural counterparts. A possible explanation could be poorer compliance to pedometer wear among Kenyan rural children. This could also be because a majority of children in urban Kenya were from poorer neighbourhoods, who have more opportunities to play or engage in PA in their neighbourhoods after school compared to their rural counterparts who may be engaged in less vigorous activities such as household chores after school. We found that lower parent education was associated with more MVPA, but only among Kenyan children. This finding is consistent with previous research in Kenya [49]. A systematic review of the correlates of PA in Sub-Saharan Africa found that higher parental education was associated with lower PA, although some studies did not observe significant associations [49]. Perceived barriers to AT were associated with less MVPA, but only in Nigeria. Even though we asked about barriers in relation to AT, concerns with issues such as traffic and personal safety may also deter PA in general. There remains a lack of quantitative studies on perceived barriers to AT and PA in Sub-Saharan Africa.

Consistent evidence from systematic reviews [53, 54] and some African studies [49, 55, 56] support the notion that active travelers are more active than motorized travelers. We found that AT was associated with higher PA in Kenya and Mozambique. However, we noted that step counts were lower among Nigerian children who engaged in AT to school. Some of these participants may have compensated for AT by being less active during the rest of the day and/or lived too close to their school for AT to make a meaningful contribution to their PA level. Another explanation could be that Nigerian children who did not engage in AT to school were accumulating more step counts in other contexts (e.g., PA at home, PA at school, active play or in AT to non-school destinations) than those who reported AT to school. However, these other potential correlates of step counts were not explored in this study. Future studies are needed to clarify the unexpected findings among Nigerian children. A previous systematic review of African studies [57] also reported inconsistent relationships between AT and PA levels. Given these findings, more research examining the association between AT and device-based measures of PA in representative samples of African children is warranted.

### Strengths and limitations

A major strength of this study is the gathering of data from children in three regions of the African continent. Additional strengths include the large sample size, the recruitment of children in rural, peri-urban, and urban areas and the use of device-based measures to assess PA. One of the study limitations is the large amount of missing data, especially for Mozambique, which limited our ability to examine the correlates of AT and PA. The use of hip-worn devices (pedometers) may under-estimate PA in activities such as cycling. However, the low rate of cycling among these children lessens the impact of this limitation on the study findings. The cross-sectional design makes it impossible to make causal inferences. Although our sample was stratified by level of urbanization within each country, classrooms were not randomly selected, and this may reduce the generalizability of the findings. Finally, the use of country-specific models of the correlates of AT, step counts, and MVPA may have increased the risk of type I error. Stratification was needed given the missing data for Mozambique and the fact that the correlates differed by country.

## Conclusion

This study provides evidence about the status and correlates of AT and PA among children living in rural, peri-urban, and urban areas in the Eastern, Western and Southern regions of Africa. Majority of children in Kenya and Mozambique engaged in AT, but across the three countries, only 23% of participants accumulated the recommended average of ≥60 min/day of MVPA. These findings underscore a clear need for PA promotion in all three countries. Low levels of PA and the potential for a decline in AT with increasing urbanization and economic growth may contribute to an increased risk of non-communicable diseases in countries where health systems are constrained by limited resources. The findings are important for health promotion officers, urban planners and policy makers as they can help inform the development of programs, plans and policies that encourage AT and PA among children. Given that the correlates of PA differed substantially between countries, a one-size-fits-all approach may be ill-advised. Practitioners and policy-makers should collaborate with researchers to identify the correlates of PA at the local level to adapt or tailor interventions to the context of implementation.

## Supporting information

S1 Table Perceived barriers to active transport. (DOCX)
