# Supplementary material for: Physical activity and active transportation behaviour among rural, peri-urban and urban children in Kenya, Mozambique and Nigeria: The PAAT Study
Source: PLoS One. 2022 Jan 21;17(1):e0262768. doi: 10.1371/journal.pone.0262768 (PMC8782337; doi:10.1371/journal.pone.0262768)
Supplement: S1 Table — (DOCX) [file pone.0262768.s001.docx]

**Supporting information file**

**S1 Table: Perceived Barriers to active transport.**

| **Item** | **1**  **Yes** | **2**  **No** |
| --- | --- | --- |
| There are too many hills along the way |  |  |
| There are no suitable walking/running or biking paths |  |  |
| The route is boring (nothing interesting to see) |  |  |
| The route does not have good lighting |  |  |
| There is too much traffic along the route |  |  |
| There are dangerous crossings |  |  |
| I get too hot and sweaty |  |  |
| No other children walk/run or bike to school |  |  |
| It’s not considered fashionable to walk/run or bike |  |  |
| I have too many things to carry |  |  |
| It is easier for my parents to drive me |  |  |
| It involves too much planning ahead |  |  |
| It is unsafe because of crime (strangers, gangs, drugs) |  |  |
| I get bullied, teased, harassed |  |  |
| There is nowhere to leave a bike safely |  |  |
| There are stray dogs or other dangerous animals |  |  |
| It is too far |  |  |
| The route is difficult to walk/run because of garbage, water or bad smells |  |  |
| The route is isolated |  |  |
| I have a disability |  |  |
| Please indicate any other challenges/barriers: | | |
